# Imaging Technologies Build Capacity and Accessibility in Phytoplankton Species Identification Expertise for Research and Monitoring: Lessons Learned During the COVID-19 Pandemic

**DOI:** 10.3389/fmicb.2022.823109

**Published:** 2022-04-14

**Authors:** Sophie Clayton, Leah Gibala-Smith, Kathryn Mogatas, Chanel Flores-Vargas, Kayla Marciniak, Maci Wigginton, Margaret R. Mulholland

**Affiliations:** ^1^Department of Ocean and Earth Sciences, Old Dominion University, Norfolk, VA, United States; ^2^New Jersey Department of Environmental Protection, Trenton, NJ, United States; ^3^Department of Biology, Old Dominion University, Norfolk, VA, United States

**Keywords:** phytoplankton, taxonomic expertise, harmful algal bloom (HAB), environmental monitoring, phytoplankton ecology, imaging cytometry, species identification, plankton

## Abstract

As primary producers, phytoplankton play an integral role in global biogeochemical cycles through their production of oxygen and fixation of carbon. They also provide significant ecosystem services, by supporting secondary production and fisheries. Phytoplankton biomass and diversity have been identified by the Global Ocean Observing System (GOOS) as Essential Ocean Variables (EOVs), properties that need to be monitored to better understand and predict the ocean system. Phytoplankton identification and enumeration relies on the skills and expertise of highly trained taxonomic analysts. The training of new taxonomic analysts is intensive and requires months to years of supervised training before an analyst is able to independently and consistently apply identification skills to a sample. During the COVID-19 pandemic, access to laboratories was greatly restricted and social distancing requirements prevented supervised training. However, access to phytoplankton imaging technologies such as the Imaging FlowCytobot (IFCB), FlowCam, and PlanktoScope, combined with open online taxonomic identification platforms such as EcoTaxa, provided a means to continue monitoring, research, and training activities remotely when in-person activities were restricted. Although such technologies can not entirely replace microscopy, they have a great potential for supporting an expansion in taxonomic training, monitoring, surveillance, and research capacity. In this paper we highlight a set of imaging and collaboration tools and describe how they were leveraged during laboratory lockdowns to advance research and monitoring goals. Anecdotally, we found that the use of imaging tools accelerated the training of new taxonomic analysts in our phytoplankton analysis laboratory. Based on these experiences, we outline how these technologies can be used to increase capacity in taxonomic training and expertise, as well as how they can be used more broadly to expand research opportunities and capacity.

## Challenges in Phytoplankton Monitoring and Training New Taxonomic Analysts

Expertise in marine phytoplankton taxonomy is largely restricted to laboratories involved in programs geared toward water quality monitoring, harmful algal bloom (HAB) surveillance, and other taxa-specific research and assessments (Stauffer et al., 2019). However, in recent years, it has become apparent that taxa-specific information is crucial to our understanding of carbon flow in aquatic systems and how this may be altered in the future due to climate change and concomitant changes in phytoplankton community structure. Indeed, the Global Ocean Observing System (GOOS) has recognized phytoplankton biomass and diversity as Essential Ocean Variables (EOVs), key variables that need to be monitored to better understand and predict the ocean system (Lindstrom et al., 2012). Surveillance and monitoring for the presence and abundance of HABs has brought many new imaging technologies to the forefront as they generate invaluable data for detecting, tracking and forecasting HAB events (Harred and Campbell, 2014; Stauffer et al., 2019). HABs are forecast to increase in frequency and duration with climate change (Najjar et al., 2010; Gobler et al., 2017; Griffith and Gobler, 2020) highlighting the need to develop better methods for early detection of HAB events and their initiation. Despite the importance of understanding taxon-specific physiological capacity and capabilities to predict how phytoplankton communities are likely to change as a result of climate change and other stressors, there has been a decline in the number of trained taxonomists and analysts (McQuatters-Gollop et al., 2017).

### The Need to Increase Capacity in Phytoplankton Species Identification Training

Skilled phytoplankton taxonomic analysts typically undergo months of closely supervised training that is generally biogeographically specific. However, since the onset of the genomics era and satellite ocean color surveillance, there has been a decline in the number of taxonomic analysts, a reduction in the number of new trainees entering the pipeline (Drew, 2011; Pearson et al., 2011; McQuatters-Gollop et al., 2017), and a shift toward molecular detection of species of interest (HARRNESS, 2005; Jewett et al., 2007; HAB RDDTT, 2008). The decline in taxonomic training has been noted by numerous US federal agencies as an impediment to long-term monitoring and tracking of HABs, and tracking changes in phytoplankton phenology and biogeography. Efforts to support training and development of taxonomic skills are urgently needed to ensure effective management, support continued surveillance for HABs, detect shifts in phytoplankton communities that may impact ecosystem structure and function, and the emergence of HAB species and shifts in the biogeography of phytoplankton communities as a result of climate change.

Taxonomic identification and enumerations of phytoplankton are crucial for early warning programs for marine and freshwater HABs, and highly trained experts are an essential part of such monitoring programs. However, classical taxonomic training can be time-consuming and costly as it requires a trained expert hours to analyze a single sample. Further, even expert taxonomists can diverge in their identifications of phytoplankton species and groups. Modern imaging technologies have been deployed on moorings or used to sample underway from ships in some regions in support of research (e.g., Martha’s Vineyard Coastal Observatory^1^; North East U.S. Shelf Long-Term Ecological Research^2^) and for early detection of HABs (e.g., Harred and Campbell, 2014) but these technologies are expensive and data are limited to either the sites where, or time periods when instruments are deployed. Although light microscopy can yield a higher taxonomical resolution of phytoplankton community composition and, in some cases, more exact species determinations than imaging methods, imaging methods have the advantage that they can produce a large number of images at higher spatial and temporal frequency, and are more geared toward the archiving and re-use of cell images. Molecular ecology tools have also been employed to identify and quantify target species for which appropriate primers are available, but this approach is generally limited to one or a few species of interest and has limited ability to produce quantitative assessments of community structure. Here we propose an alternative method for training taxonomic analysts that combines the use of images generated by automated imaging systems with more traditional techniques, to accelerate training and expand capacity. Additionally, we highlight how image libraries generated through the use of imaging tools can be used to harmonize species identification across laboratories, and provide a spatially− and temporally resolved data archive of phytoplankton community composition that can be re-interrogated over time as research questions evolve.

### Lessons Learned From the COVID Lockdown: An Accidental Experiment Leading to Adaptations to Workflow

The Phytoplankton Analysis Laboratory at Old Dominion University (referred to hereafter as the ODU Phyto Lab) has been in operation since the early 1980s and undertakes a range of taxonomic analyses in support of research projects as well as local, state and federal agencies involved in the Chesapeake Bay restoration, and shellfish and public health monitoring in coastal and freshwater bodies in the Commonwealth of Virginia (VA). Although the ODU Phyto Lab is managed by full-time experts in phytoplankton taxonomic analysis, it also relies on undergraduate and graduate student interns rotating through the laboratory who must be trained in phytoplankton taxonomy and microscopy. Trainees are expected to conduct microscope analysis on samples collected from monitoring stations associated with a long-term monitoring project through the Chesapeake Bay Program and shellfish growing areas within waters of Virginia. This requires species-level understanding, with special attention paid to a suite of seasonal bloomers, including the potentially toxigenic HABs *Pseudo-nitzschia* spp. and *Dinophysis* spp. Up until the onset of the COVID-19 pandemic, this training involved laboratory staff working at close quarters with trainees and having access to microscopes and slide preparation materials in the laboratory.

An Imaging FlowCytobot (IFCB; Olson and Sosik, 2007) was acquired in 2019, and samples being collected as part of a local time series site (Mulholland et al., 2018) were used to begin to build an image database and prepare the instrument for deployment. When the ODU Phyto Lab’s regular taxonomic analysis training model was interrupted by the COVID-19 pandemic, the image library produced by the IFCB was leveraged to implement and test different training and analysis methods and tools. During the COVID-19 pandemic, many laboratories including the ODU Phyto Lab, were shut down and/or required to practice social distancing. As a result, taxonomic training using microscopes with trainer and trainee in close proximity in the laboratory became impossible despite the need for continuity in the monitoring work on which state and federal entities depend. It became necessary to develop new, socially distanced ways to continue the ODU Phyto Lab’s monitoring, research, and training activities that had until then relied solely on the ability of staff to work collaboratively on shared microscopes.

Although access to the laboratory during the early part of the pandemic was restricted, it was still possible for a single individual to collect samples in the field, run those samples through an imaging instrument in the laboratory, and process and upload the images to EcoTaxa (Picheral et al., 2017^3^), an open access web application for the taxonomic annotation of images of plankton. The water samples collected and images processed prior to the COVID-19 lockdown also provided a large existing image bank that could be used for training while field sampling was suspended. Using this image bank, students were trained to identify a subset of the taxa present in the phytoplankton community for their individual projects. This approach proved to have many advantages over the more traditional microscope-based training methods which requires working at close quarters in the laboratory, direct supervision by the trainer, and complete identification and enumeration of the phytoplankton community within a sample before it is discarded. Here we outline how this approach might, in conjunction with traditional microscopy methods, modernize taxonomic training and phytoplankton analyses to build capacity in taxonomic expertise for research, speed up the detection of and response to HAB events, expand monitoring capacity by increasing the frequency and spatial coverage of sampling, and create databases that can be re-interrogated as our understanding of phytoplankton communities evolve and climate changes.

## Short Overview of Technologies and Tools Used in This Case Study

Several cell imaging instruments have been developed over the last decade and a handful have matured to the point where they are commercially available and routinely used as part of HAB and phytoplankton monitoring programs and research. These technologies have been reviewed in detail elsewhere (Lombard et al., 2019; Stauffer et al., 2019), but here we will briefly describe three imaging instruments that have been in use by the ODU Phyto Lab since January 2020, and that form the basis of the recommendations outlined here. The ODU Phyto Lab has access to an IFCB; several PlanktoScopes, low-cost imaging microscopes which have been built in-house (Pollina et al., 2020^4^); and a FlowCam (Sieracki et al., 1998) loaned to the ODU Phyto Lab for 3 months through the manufacturer’s grant program. The IFCB was used to image cells in raw seawater samples, whereas the PlanktoScopes and FlowCam were used to generate images from both preserved (generally using Lugol’s) and unpreserved water samples.

Images collected using the IFCB, FlowCam and PlanktoScope were uploaded to EcoTaxa, an open access online web application for the taxonomic annotation of images of plankton, and annotated/classified by the ODU Phyto Lab and two undergraduate interns, taxonomic analysts in training. Images collected using the IFCB are processed using a set of publicly available MATLAB-based tools,^5^ which segment the images into individual regions of interest (ROIs) and generate a set of geometric parameters based on the ROIs (e.g., major axis length, minor axis length). Images produced using a PlanktoScope are processed in a similar way using MorphoCut, a publicly available Python-based analysis pipeline which can be run directly on the PlanktoScope’s onboard Raspberry Pi computer to segment images into ROIs and generate descriptive geometric parameters.^6^ FlowCam provides proprietary software called VisualSpreadsheet for analyzing and extracting data from FlowCam images. Although previous versions of VisualSpreadsheet allowed for FlowCam images to be uploaded to EcoTaxa, the new version of the software uses a very different file structure, and so FlowCam images cannot be directly uploaded for analysis, however it is possible to work around this limitation using MorphoCut. After sample analysis, images generated by the IFCB, FlowCam, and PlanktoScope, their geometric characteristics and associated metadata can be uploaded to EcoTaxa for annotation, classification and validation. Although other similar pipelines exist for image annotation, classification and validation, the main advantage of EcoTaxa for use during the pandemic lockdowns was the fact that it is a web-hosted application that can be accessed from anywhere with an internet connection. As a result it was not necessary for laboratory personnel or student interns to work in shared spaces, use laboratory computers or even to log in to institutional servers to work with or train on the image data. EcoTaxa also allows for the tracking of annotations by different users, allowing for a highly iterative approach to both training and sample analysis, as feedback and corrections to annotations could be done asynchronously as needed. Another big advantage of the web-based application was that training could be done synchronously but remotely, with trainers and trainees communicating using video conferencing tools while accessing the same image bank without needing to be physically co-located. This combination of imaging instruments and an easily accessible online platform are the basis of the training model described below.

## An Imaging Technology-Supported Model for Species Identification Training

Using images and online sharing tools, we developed a set of guidelines for using technologies to train students in phytoplankton species identification, following a traditional sequence. Experienced analysts within the ODU Phyto Lab identified a set of images from an ongoing (or current) investigation for a select group of taxa that occured in large numbers within the image collection. These categorized images, along with traditional taxonomic reference materials, were provided to trainees to allow them gain familiarity with the morphological features used to identify those specific taxa. Initially trainees were taught to recognize cell shapes, leading to the initial classification of functional groups, e.g., classify cells as dinoflagellates or diatoms. Subsequently trainees were gradually taught to recognize and classify taxa within a given functional group, e.g., centric vs. pennate diatoms. Experienced analysts and trainees, using Zoom and share screen functions with the EcoTaxa project page in view, reviewed targeted taxa structure and how to apply traditional microscope identification skills to reviewing the images in the EcoTaxa project. The trainees were then assigned the task of finding further images of those taxa in the project’s image collection. The trainers could then check the trainees’ annotations to make sure that they were identifying the cells in the images correctly. When difficulties arose, experienced analysts were able to quickly respond to questions, and teach the trainees how to better differentiate between similar looking groups either by real-time communication *via* Zoom or emailing images for asynchronous responses. In addition, as the trainee would scan the image collection looking for their targeted taxa, they became familiar with other groups of organisms and were able to increase the number of taxa they could accurately and correctly identify. Almost more importantly, trainees were able to quickly identify a cell or group of cells that did not fall into a group they were familiar with, set them aside into a single temporary holding category area, and use their newly acquired skills to independently use the resources available to them to determine their correct taxonomic group. This skill developed much more rapidly with our “COVID experiment” trainees than had ever occurred with traditionally trained students in the 6 years prior, within days or a few weeks as opposed to many weeks or months.

During this time, ODU Phyto Lab, trainees were tasked with identifying particular subgroups of phytoplankton taxa while also contributing to annotating images of other taxa they were familiar with, thereby enhancing both their taxonomic analysis expertise and the skill of machine learning algorithms used for automated classification. For example, after gaining a broad but superficial mastery of the shapes of different phytoplankton functional groups, trainees were then given more in-depth training for only one of the those functional groups, with some trainees focusing on dinoflagellates, others on centric diatoms, and yet others on pennate diatoms. In this way, the identification of phytoplankton in samples was distributed across trainees and laboratory personnel with more senior and experienced taxonomists able to act as a check on trainees’ image classifications. Trainees did not need to have a broad taxonomic knowledge to start contributing meaningfully to the identification work because images could be revisited as machine learning algorithms were refined. Anecdotally, we saw shorter training periods when incorporating imaging into our traditional training method, with trainees trained with imaging able to transition to working and analyzing microscopy samples independently after around 3 months, about half the usual 6 month training period.

**FIGURE 1:**
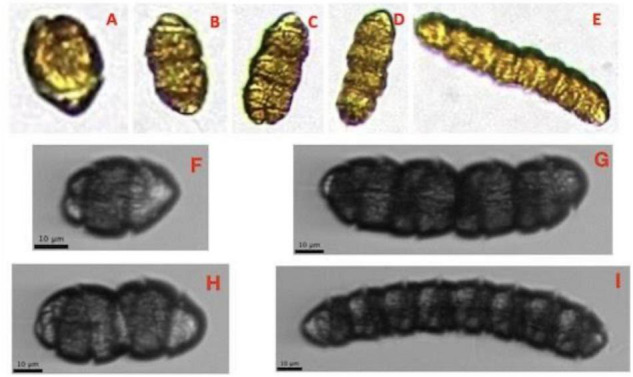
Images of *Margalefidinium polykrikoides* generated from different imaging instruments. The top row **(A–E)** shows images of single and multiple cell chains produced by a PlanktoScope from unpreserved samples. The second and third rows **(F–I)** show images of single and multiple cell chains produced by an IFCB from unpreserved samples. The water samples were all taken from the lower Chesapeake Bay in August 2020 and 2021.

As outlined above, the use of imaging technology for species identification training is an iterative process that relies on the images being collected, some of them annotated (e.g., identified and labeled with an associated taxonomic classification), analyzed using a machine learning classification algorithm and then archived, allowing for them to be revisited at a later date. When working with samples under a microscope, unless images are collected manually, no visual record remains after analysis. Thanks to the automated saving and archiving of phytoplankton images by imaging technologies, their use allows for some specific and important differences and advantages over light microscopy for training:

1. People can be trained quickly and remotely, as compared with traditional microscopy techniques. This represents a real potential for building capacity in the field. Accuracy can be improved through online resources and image libraries.
2. Training does not need to be “all at once,” that is, trainees can learn to identify specific taxa or functional groups without needing to be familiar with all of the taxa that might be found in a sample. Trainees can contribute to a laboratory’s analysis work at an earlier stage of their training.
3. Using a web-based application such as EcoTaxa, user annotations and validations are tracked, so feedback between the trainer and trainee can be asynchronous, as the trainer does not need to check a physical sample before it can be discarded.
4. Curated image libraries for reference and training can be built and added to over time (as has been done with data from the IFCB deployment at MVCO, Sosik et al., 2015). Regional and international image libraries can be developed in collaboration with other laboratories around the world to ensure consistency in identification and to help standardize taxonomy regionally and globally. Images from any platform, including light microscopy, can (and should) be included in such libraries.
5. Image libraries will include images of cells from different orientations as well as different life stages (e.g., resting cysts, multiple cell chains, Figure 1). This can lead to new information and assist in harmonized species identifications and estimations of biovolumes (Orr et al., 2021).

In summary, with imaging techniques, sample imaging and analysis can be asynchronous, and trainees do not need to learn the entire phytoplankton species composition all at once. They can focus on certain taxa or groups leaving the full dataset to be interrogated iteratively later. Mastering species identification can take years and this method of training allows for students to reliably identify specific constituents of the phytoplankton community rather than the full taxonomic range that might be encountered, thereby allowing trainees to contribute to phytoplankton community composition evaluations of samples in meaningful ways immediately. We also reiterate that, in our admittedly limited case study, we found that including imaging in training resulted in an appreciable reduction in that time needed for a trainee to become proficient enough in species identification to work independently.

## Expanding Harmful Algal Bloom Monitoring and Phytoplankton Research With Imaging Technologies

Many of the advantages of using imaging for phytoplankton species identification training can also be leveraged to enable advances in phytoplankton ecology research and HAB surveillance and monitoring applications. In both cases, research and monitoring are hampered by a scarcity of taxonomically resolved field data in time and space. Because field samples can be processed and imaged relatively quickly using the imaging platforms described above, live cells from many samples can be imaged and the data stored for analysis at a later date, thereby increasing sample throughput. There is a tradeoff between resolution, which is generally better using light microscopy, and sample throughput, which is higher for imaging instruments. However we do note that the ability to quickly image live cells does have the advantage that taxa such as coccolithophores or naked flagellated species that might degrade or be altered when administered preservatives such as Lugol’s solution or formalin (Throndsen, 1978) can be captured by imaging instruments. As a result, the wider adaptation of imaging technologies will greatly increase the volume of data available for a wide range of research and monitoring activities. Thanks to the large number of images produced, machine learning algorithms can be trained and used to automate the identification of phytoplankton cells allowing for a much greater number of samples to be enumerated than by microscopy alone. Further, through the sharing of image databases, taxonomic identifications can be harmonized regionally and globally.

The development of low-cost imaging instruments such as the PlanktoScope which we have described above, as well as other platforms such as the HABscope^7^ which has been designed for use by trained volunteers, is opening up the possibility of generating near real-time cell counts directly from field sampling locations. These low-cost instruments can be provided to stakeholders such as agency field staff or shellfish farmers who can run samples in the field and upload the data to a central repository such as EcoTaxa, assuming that they have internet access. The resulting images are then classified using machine learning models trained on locally generated image libraries, and some may also be inspected remotely by expert taxonomists to ensure the performance of the classification algorithm. This could allow for a significant expansion of HAB monitoring, generating higher frequency data in both time and space, and speeding up the time between sample collection and identification. HABscope has already done this for monitoring and detection of *Karenia brevis* in the Gulf of Mexico (Hardison et al., 2019).

Establishing and maintaining image libraries either regionally or globally will be key to capitalizing on many of the novel advantages of plankton imaging technologies. We envisage that such libraries will allow for regional tracking of climate change impacts on specific phytoplankton communities, for example, enabling the detection and tracking of invasive species. Similarly, image libraries could be used to identify, assess, and track temporal or regional physiological differences (e.g., cell major and minor axis lengths, mean cell sizes, biovolume, chain length, etc.) among populations and communities. This will be of particular relevance to the development of ecological and biogeochemical models, whose performance can be difficult to assess due to lack of data on specific taxa of interest (e.g., HAB prediction models), and difficulties in converting cell densities (e.g., cells L^–1^) reported from microscopic enumeration to biomass concentrations (mmol C m^–3^) used in ecological models.

## Conclusion

In this paper we have outlined ways in which imaging tools and online data sharing platforms can be used to increase capacity in phytoplankton species identification expertise, and expand monitoring capacity, summarized in Table 1. The experience that trainees obtained using the image library prior to being introduced to the phytoplankton community in samples on the microscope allowed them to reach a level of proficiency rapidly, thus increasing workflow and laboratory productivity. Although anecdotal, our experience has shown that imaging technologies can be used to shorten by up to half the period of training required before trainees can make meaningful contributions to generating robust species identifications for monitoring and research purposes. Imaging technologies and open online sharing platforms allowed us to completely and successfully change our species identification training model during the height of the COVID-19 pandemic and continue monitoring for state and local agencies as well as driving new research regarding the life stages of dinoflagellates. As a result of the pandemic experience, we have identified ways in which imaging technologies can be used to accelerate and increase efficiency in phytoplankton species identification training, as well as enhancing sample throughput and HAB monitoring, and facilitating regional and global inter-comparison studies, and tracking regional and global intraspecific variability.

**TABLE 1:** Summary table of the respective advantages of imaging and microscopy for different applications requiring taxonomic identification of phytoplankton.

	APPLICATION		
	Species Identification Training	HAB Tracking and Monitoring	Phytoplankton Ecology
**IMAGING**	• Speeds up the training process. • Trainees can work with samples non-destructively. • Trainees can be tasked with identification of a subset of species of interest. • Trainers and trainees can interact and work remotely and asynchronously.	• Higher frequency of sampling in time and space is possible. • Enables inter-calibration between regional and global laboratories. • Allows rapid counts of a small suite of target species without losing information on other species present. • Allows for real time results to inform regulatory actions even if field staff or citizen scientists are not trained taxonomic analysts (i.e., through automated classification of images).	• Can generate large image libraries which enables regional and global inter-comparisons. • Images can be archived for re-examination and analysis at a later date. • Allows for easier estimation of cell size and biovolume, useful for model validation and comparison. • Images cells from multiple orientations allowing more accurate estimates of biovolume.
**MICROSCOPY**	• Allows for better optical resolution and precise focus to investigate morphological features of a cell that occur on different focal planes. • Allows for the use of specialized methods, such as fluorescent dyes to facilitate identification based on specific morphological features.	• Allows for detection of emerging HAB taxa that may be unexpected in the area or not previously detected. • Allows for better optical resolution and precise focus to investigate different focal planes. • Allows for the use of specialized methods, such as fluorescent dyes.	• Allows for more reliable enumeration of rare species as sample volumes may be larger. • Allows for finer distinction between similarly structured species. • Allows for better resolution of small celled taxa (<5 μm) that are mostly missed by imaging instruments.

Although we have outlined many advantages of using imaging technologies for phytoplankton species identification training and research, we do not advocate that imaging tools should completely replace microscopy. There are particular applications that imaging is not well suited for, in particular for achieving high taxonomic resolution in identifications, which light microscopy allows thanks to the higher optical resolution and ability to scrutinize the cell at different focal levels. Imaging is also poorly suited to identifying and enumerating picophytoplankton and nanophytoplankton that are smaller than ∼ 5 μm and not always detected by imaging instruments. While imaging coupled with automated classification tools can process a larger number of samples than microscopy, microscopy has been shown to achieve higher taxonomic resolution (Álvarez et al., 2014). We also recognize that not all laboratories have the resources required to implement the use of imaging in their training or analysis work, due to the expense of imaging instruments such as the IFCB or FlowCam. These instruments, although useful for generating very large quantities of images, are not required to apply some of the workflow described here. The important step is to generate images that can then be shared with trainees in some way. This could be achieved with light microscopes equipped with cameras (which are commonly found in laboratories), or by using cheap open-source instruments such as the PlanktoScope imaging microscope that can be built by non-experts for < $1,000 each. Finally, we reiterate that we do not advocate that imaging technologies should replace microscopy, but rather that both can be used in concert to expand capacity in taxonomic analysis and through that expanded capacity, drive new advances in the fields of phytoplankton taxonomy and ecology.

## Data Availability Statement

The original contributions presented in the study are included in the article/supplementary material, further inquiries can be directed to the corresponding author.

## Author Contributions

SC, MM, and LG-S contributed to the conception of the manuscript. SC, LG-S, KMo, and MM drafted the manuscript. All authors contributed to manuscript revision, read, and approved the submitted version.

## Conflict of Interest

The authors declare that the research was conducted in the absence of any commercial or financial relationships that could be construed as a potential conflict of interest.

## Publisher’s Note

All claims expressed in this article are solely those of the authors and do not necessarily represent those of their affiliated organizations, or those of the publisher, the editors and the reviewers. Any product that may be evaluated in this article, or claim that may be made by its manufacturer, is not guaranteed or endorsed by the publisher.

## Footnotes

1
https://ifcb-data.whoi.edu/timeline?dataset=mvco

2
https://nes-lter.whoi.edu/data/

3
http://ecotaxa.obs-vlfr.fr

4
https://www.planktoscope.org/

5
https://github.com/hsosik/ifcb-analysis/wiki

6
https://github.com/morphocut/morphocut

7
https://habscope.gcoos.org/
